# Ethanol and Protein from Ethanol Plant By-Products Using Edible Fungi *Neurospora intermedia* and *Aspergillus oryzae*

**DOI:** 10.1155/2015/176371

**Published:** 2015-11-23

**Authors:** Veronika Bátori, Jorge A. Ferreira, Mohammad J. Taherzadeh, Patrik R. Lennartsson

**Affiliations:** ^1^Swedish Centre for Resource Recovery, University of Borås, 501 90 Borås, Sweden; ^2^Faculty of Forestry, University of West Hungary, Sopron 9400, Hungary

## Abstract

Feasible biorefineries for production of second-generation ethanol are difficult to establish due to the process complexity. An alternative is to partially include the process in the first-generation plants. Whole stillage, a by-product from dry-mill ethanol processes from grains, is mostly composed of undegraded bran and lignocelluloses can be used as a potential substrate for production of ethanol and feed proteins. Ethanol production and the proteins from the stillage were investigated using the edible fungi *Neurospora intermedia* and *Aspergillus oryzae*, respectively. *N. intermedia* produced 4.7 g/L ethanol from the stillage and increased to 8.7 g/L by adding 1 FPU of cellulase/g suspended solids. *Saccharomyces cerevisiae* produced 0.4 and 5.1 g/L ethanol, respectively. Under a two-stage cultivation with both fungi, up to 7.6 g/L of ethanol and 5.8 g/L of biomass containing 42% (w/w) crude protein were obtained. Both fungi degraded complex substrates including arabinan, glucan, mannan, and xylan where reductions of 91, 73, 38, and 89% (w/v) were achieved, respectively. The inclusion of the current process can lead to the production of 44,000 m^3^ of ethanol (22% improvement), around 12,000 tons of protein-rich biomass for animal feed, and energy savings considering a typical facility producing 200,000 m^3^ ethanol/year.

## 1. Introduction

**Figure 1:**
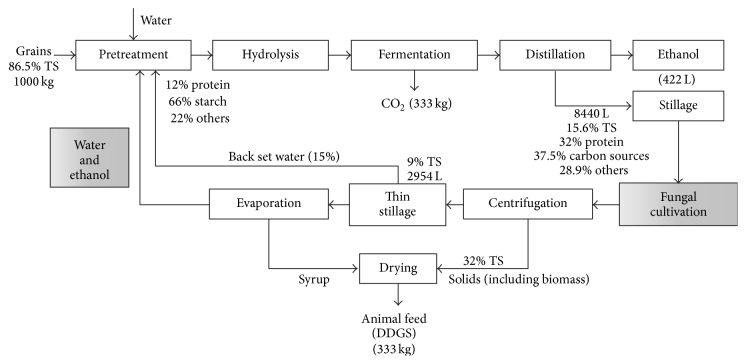
Overall industrial scheme of production of ethanol from grains with process modifications via inclusion of the process of ethanol production from whole stillage by* N. intermedia* (highlighted boxes). The indication of TS, production volumes, and composition relate to the normal process without inclusion of the process of ethanol production from whole stillage.

Currently bioethanol is the dominant biofuel in the transport sector. Corn and sugarcane are the most widely used feedstock in the bioethanol industry, following other materials such as wheat and crop roots [1]. In addition to ethanol, dry-mill starch-based processes also produce DDGS (Distillers Dried Grains with Solubles), a source of protein for animal feed from the fermentation residues. After the fermentation and distillation of ethanol, the slurry leaving the bottom of the column (whole stillage) contains the oil, protein, fibre, and other unfermented components of the grains and yeast cells [2]. Up to 20 litres of whole stillage is produced per litre of ethanol from corn or wheat grains [3], which means a global production of more than a billion tons per year. Whole stillage undergoes a centrifugation step to produce a liquid fraction (thin stillage), 15% or more of which is used as backset water and a solid fraction (wet distiller's grains). The syrup resulting from the series of evaporations of the remaining thin stillage is dried with the wet distiller's grains to produce DDGS (Figure 1) [2]. In 2011, the ethanol sector produced ca. 86 million metric tons of ethanol and 68 million tons of DDGS [4, 5]. Reasonably, the revenues from the DDGS play an important role for the overall process economics [1] due to its large volume, which make up for the relatively low value which also presents an opportunity, since relatively small improvements can have a large impact. The large economic importance of the DDGS also puts a limit on the potential solutions, since the feed quality of DDGS cannot be compromised. In practice, this means that any retrofitting must fulfil feed quality standards, which also includes any introduced microorganism [6]. A potential solution is to use edible filamentous fungi to produce ethanol and protein-rich fungal biomass.

Filamentous fungi are known to be able to produce a wide array of enzymes enabling them to degrade complex substrates. This is one of the reasons that some filamentous fungi such as* Neurospora intermedia* and* Aspergillus oryzae* are used for production of fermented food such as oncom [7] and enzymes for feed, beverage, textile, and paper and pulp industries, respectively [8]. Many fungi also have the ability to utilize sugars such as xylose that baker's yeast (*Saccharomyces cerevisiae*) cannot assimilate. These advantages have sparked a research interest in developing new processes based on filamentous fungi. Related examples include fungal biomass production from spent sulphite liquor [9] and corn-based thin stillage [10] with* Rhizopus *sp. and ethanol and fungal biomass production from wheat-based thin stillage with* N. intermedia* [11].* N. intermedia* was found to produce the highest amount of ethanol (5 g/L) in comparison to other ascomycetes such as* A. oryzae*,* Fusarium venenatum*,* Monascus purpureus*, and the zygomycete* Rhizopus* sp. and its biomass was 56% (w/w) crude protein. Implemented in a bioethanol process both production of ethanol and fungal biomass from the residues have the advantage of being relatively easy to separate through filtration and evaporation. The latter is already in use in the drying process for the DDGS, with the condensate being sent back to the beginning of the ethanol production process. The ethanol produced by* N. intermedia* would therefore only influence the overall industrial process by increasing the final ethanol concentration in the mash and would be separated in the normal distillation step.

Whole stillage has a theoretically higher potential for ethanol production than thin stillage as a higher solid content is available. This solid content consists mainly of undegraded bran if whole-meal wheat is used as feedstock and is thus a lignocellulosic material. Ethanol production from whole stillage could therefore not only result in even more ethanol being produced from current resources, but also serve as an important step towards second-generation ethanol. Information regarding production of ethanol from whole stillage is currently lacking in the scientific literature. Being of lignocellulosic origin, addition of enzymes might enhance the production of ethanol via degradation or increase of the degradation rate of complex substrates that* N. intermedia* might naturally assimilate. The commercial available cellulase has widely been used for conversion of cellulose to fermentable sugars [12–14]. However, the high solid content of the whole stillage hampers a clear separation of the produced fungal biomass if a purified protein-rich fungal product for feed applications is desired. A two-stage cultivation including ethanol production followed by biomass production after separation of the suspending fibres might represent an alternative.

In this study, the potential of whole stillage as a substrate for production of ethanol was investigated. Special focus was on the influence of enzyme loading and different whole stillage fractions on ethanol production. A comparison of production of ethanol from whole stillage between* N. intermedia *and baker's yeast* S. cerevisiae* was also carried out in order to investigate the need to introduce a new microorganism in the established industrial process. Furthermore, a novel strategy based on a two-stage cultivation was also investigated including ethanol production by* N. intermedia* under simultaneous saccharification and fermentation followed by production of protein-rich biomass by* Aspergillus oryzae* after the medium had been distilled and sieved.

## 2. Materials and Methods

### 2.1. Substrate

The whole stillage, originating from a dry-mill bioethanol production process based mainly on whole-meal wheat, was kindly provided by Lantmännen Agroetanol (Norrköping, Sweden). The whole stillage, originated from a single batch, was autoclaved for 30 minutes at 121°C and stored at 4°C until use.

### 2.2. Microorganisms

The ascomycetes* Neurospora intermedia* CBS 131.92 and* Aspergillus oryzae* var.* oryzae* CBS 819.72 (Centraalbureau voor Schimmelcultures, Netherlands) were used and maintained on potato dextrose agar (PDA) medium containing 20 g/L glucose, 15 g/L agar, and 4 g/L potato extract. New plates were prepared via incubation at 30°C for 3-4 days and stored at 4°C. Spore solutions for inoculation were prepared by flooding each plate with 20 mL sterile distilled water and disposable plastic spreaders were used to release the spores into the liquid. Spore concentration was determined by using a counting chamber. Ethanol Red (Fermentis, France), a specially selected strain of* Saccharomyces cerevisiae* for industrial ethanol production, was also used and maintained on yeast peptone dextrose agar (YPDA) medium, containing 20 g/L glucose, 20 g/L agar, 20 g/L peptone, and 10 g/L yeast extract. New plates were prepared via incubation at 30°C for 2 days and stored at 4°C. For inoculation one yeast colony was added to the medium.

### 2.3. Cultivation in Shake-Flasks

Cultivation was, unless otherwise noted, carried out in cotton plugged 250 mL wide-necked Erlenmeyer flasks containing 150 mL of medium autoclaved at 121°C for 20 min. A water bath was used to maintain the temperature at 35°C and continuous shaking at 125 rpm. Each flask was inoculated with 3 mL of* N. intermedia* spore solution, resulting in 1.14  (±0.53) × 10^5^ spores/mL. Stillage was adjusted to pH 5 prior to cultivation with 10 M NaOH and 6 M HCl. Samples were taken from the fermentation broth at predetermined times and centrifuged at 10,000 ×g for 10 minutes. The supernatant was kept at −20°C until analysis. Unless otherwise stated, cultivation was done in duplicate.

#### 2.3.1. Whole Stillage

Undiluted whole stillage was treated with 0, 1, 5, and 10 FPU Cellic Ctec2/g SS (Novozymes, Denmark). Enzyme was added along with spore solution and cultivation was carried out for 120 h. The enzyme activity was 94 FPU/mL.

#### 2.3.2. Fractionation

Whole stillage was divided into 3 fractions, large particles by sieving, small particles by centrifugation using a semicontinuous centrifuge (CEPA, Germany) at 29,000 ×g and a flow of 5 L/h, and the remaining liquid phase. The solid particles were washed with distilled water and were finally resuspended in distilled water to achieve the same concentration as in the whole stillage. Cultivation in triplicate was carried out with* N. intermedia* for 120 h, both with and without addition of 1 FPU Cellic Ctec2/g SS.

#### 2.3.3. Comparison with* S. cerevisiae*

Cultivation of whole stillage was also carried out using* S. cerevisiae*, both as monocultures and as cocultures with* N. intermedia*. Flasks that were inoculated with monocultures were done with and without enzyme treatment. Enzyme load was 1 FPU/g SS. Cultivation was carried out for 120 h.

#### 2.3.4. Carbohydrate Assimilation

Carbohydrate assimilation was examined using semisynthetic medium containing 7.5 g/L (NH_4_)_2_SO_4_, 3.5 g/L KH_2_PO_4_, 1.0 g/L CaCl_2_·2H_2_O, 0.75 g/L MgSO_4_·7H_2_O, 10 mL/L trace metal solution, 5 g/L yeast extract, and different carbon sources. Carbon sources were cellobiose, starch, cellulose (Avicel), and xylan (30 g/L, resp.). Cultivation time was 144 h for cellobiose and starch and 166 h for Avicel and xylan.

### 2.4. Cultivation in a Bioreactor

Whole stillage was cultivated in a 2.5 L continuous-stirred tank reactor (CSTR) (Biostat A, B. Braun Biotech International, Germany). The cultivation was carried out in two 72 h stages. In both cases temperature was controlled and kept at 35°C, stirring at 250 rpm, air flow rate at 25 L/h, and pH at 5.0 with addition of 2 M NaOH or 2 M H_2_SO_4_. The 24 h inoculums were prepared in 250 mL wide-necked Erlenmeyer flasks containing 100 mL of yeast peptone dextrose medium (YPD) composed of 20 g/L glucose, 5 g/L peptone, and 5 g/L yeast extract. Incubation was carried out as described before. In the first stage of cultivation, 1 FPU cellulase/g SS and the inoculum of* N. intermedia* (dry weight: 1.82 ± 0.31 g/L) were added to 2.01 (±0.01) kg of whole stillage. After 72 h cultivation, ethanol in the broth was separated from the remaining liquid using a rotary evaporator (Laborota 20 eco, Heidolph, Germany) at 110°C, 20 rpm, and 400 mbar. The evaporated water was compensated for by addition of distilled water and the medium was sieved. In the second stage of the experiment, 1 L of the obtained liquid was inoculated with previously grown* A. oryzae* biomass (dry weight: 2.70 ± 0.25 g/L). Cultivation was carried out in duplicate.

### 2.5. Analytical Methods

Stillage suspended solid content was determined by washing and vacuum filtration with Büchner funnel and Whatman (Cat. number 1001-070) filter paper, followed by drying at 70°C for 24 hours. Total solid and dry content were determined by drying the material in oven at 70°C for 24 h, achieving constant weight. Harvested biomass was dried following the same protocol.

Crude protein content was determined according to the Kjeldahl method applying digestion, distillation, and acid-base titration using an InKjel P digestor and a behrotest S1 distiller (behr Labor-Technik, Germany). Digestion was carried out by adding 20 mL of 98% (v/v) H_2_SO_4_, antifoam, and KT1 tablets (Thompson & Capper Ltd., UK) to 0.51 ± 0.04 g material for a total duration of 100 minutes at 100% power (of which 10 min was for heating up the system). Digestion was followed by neutralization of the digested solution with 32% (w/w) NaOH and distillation for 5 min. The distillation vapour was trapped in 50 mL of 4% H_3_BO_4_. Final titration was carried out with 0.100 M of HCl until pH 4.6. A factor of 6.25 was used to calculate the crude protein content.

Spore concentration was determined using a Bürker counting chamber. The spores were counted in 144 E-squares (1/250 *μ*L) and a final concentration of solution was calculated.

For identifying and quantifying different components of the broth samples high performance liquid chromatography (HPLC) (Waters 2695, Waters, Milford, USA) analysis was used. Acetic acid, ethanol, glucose, glycerol, lactic acid, and xylitol were analysed using an analytical ion exchange column based on hydrogen ions (Aminex HPX-87H, Bio-Rad, USA) operated at 60°C with 0.6 mL/min of 5 mM H_2_SO_4_ as eluent. Arabinose, galactose, glucose, mannose, and xylose were analysed using a lead(II)-based column (HPX-87P, Bio-Rad) with two Micro-Guard Deashing (Bio-Rad) precolumns operated at 85°C with 0.6 mL/min ultrapure water as eluent. A UV absorbance detector (Waters 2487), operating at 210 nm wavelength, was used in series with a refractive index (RI) detector (Waters 2414).

Structural carbohydrates of the solid biomass samples were prepared for determination according to NREL/TP-510-42618 [15]. Total amount of dissolved carbohydrates was determined according to NREL/TP-510-42623 [16]. The cellulase Cellic Ctec2 activity was measured according to NREL/TP-510-42628 [17] using a Biochrom Libra spectrophotometer (Biochrom, UK).

All analyses were carried out in duplicate and reported intervals and error bars are ±2 standard deviations, unless otherwise noted.

### 2.6. Statistical Analysis

Statistical analysis of the data obtained from the enzyme loading experiments was performed using the software package MINITAB 17. Results were analysed with ANOVA (analysis of variance) using general linear models with a 95% confidence interval.

## 3. Results and Discussion

The economical robustness of established biorefineries can greatly rely on its intrinsic capacity to further improve the process. The strategy normally involves the valorisation of side streams via production of value-added chemicals. The process of a typical ethanol plant (200,000 m^3^ of ethanol/year) from corn or wheat grains can give rise to up to 4 million m^3^ of whole stillage [3]. Moreover, the additional steps (Figure 1) in order to produce the DDGS including centrifugation of the whole stillage, evaporation of the thin stillage, and drying are responsible for a large fraction of the overall process energy expenses [18]. Thin stillage, originating from a whole-wheat ethanol process, has been successfully investigated for further ethanol production [11] using* N. intermedia*. Further studies have shown that dissolved saccharides and sugar polymers (e.g., xylan) present in the suspended solids are among the carbon sources that* N. intermedia* can assimilate and convert to ethanol [19]. Considering that the whole stillage has not gone through a first centrifugation step yet, its suspended solid content is higher and so is its potential for production of ethanol. Moreover, the changes and low investment needed for the inclusion of production of ethanol by* N. intermedia* from thin stillage would be similar to those needed if the cultivation medium is whole stillage (Figure 1).

From the parameters evaluated and presented in Table 1, the stillage used in this work was composed of around 60 g/L of potential carbon sources in addition to its relevant amount of crude protein. Glucose- and xylose-based dissolved saccharides and the polymers glucan and xylan, all potential substrates for ethanol production, made up around 38% and 14% of the measured carbon sources, respectively. The use of enzymes in order to increase the ethanol production from lignocellulosic substrates has been widely investigated and demonstrated in the literature [20]. Therefore, research on the effect of cellulase complex Cellic Ctec2 addition on ethanol production from whole stillage using* N. intermedia* was carried out in this work. A performance comparison between the widely used baker's yeast* S. cerevisiae* and* N. intermedia* towards ethanol production was also carried out. Research on production of a second value-added product, namely, fungal biomass, for feed applications was further studied by applying a two-stage cultivation strategy.

### 3.1. Effect of Cellulase Loading on Ethanol Production

**Figure 2:**
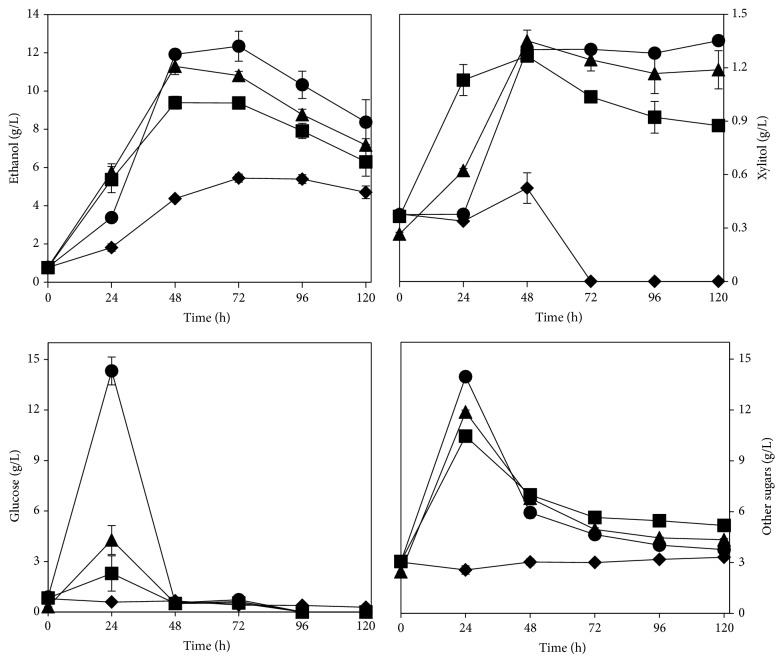
Profiles of ethanol, xylitol, glucose, and other sugars during cultivation of* N. intermedia* in whole stillage with 0 (diamonds), 1 (squares), 5 (triangles), and 10 (circles) FPU of cellulase per g of suspended solids. Error bars represent two standard deviations.

Undiluted stillage was treated with different loadings of cellulase and cultivation was carried out in simultaneous saccharification and fermentation mode. As depicted in Figure 2, the addition of enzyme led to clear improvements in the maximum ethanol production in comparison to that when no enzyme was added (*p* value = 0.000). The highest amount of ethanol (11.6 ± 0.8 g/L) and the highest production rate (232 ± 6 mg/L/h) were achieved when the highest enzyme loading was used. However, the most striking differences were observed between the absence of enzyme and its use at 1 FPU/g SS where an increase of 85% and 98% was achieved in the ethanol production and production rate, respectively. Moreover, remarkable differences were observed when cellulase was added to the medium regarding the release of sugars (glucose and other sugars), production of xylitol, and carbon source consumption patterns during cultivation. Within the sampling time used in this work, up to 14 g/L of glucose was detected when the highest concentration of enzyme was used. Moreover, up to 160% increase in xylitol production and up to 324% increase in the release of other sugars were detected. Total consumption of acetic acid and no significant changes in the glycerol concentration were observed at all tested conditions. The consumption of xylitol and of the other sugars showed a decreasing and increasing trend, respectively, at gradually higher enzyme loadings. The xylitol production indicates that* N. intermedia* had consumed xylose and that its conversion occurs probably via the general fungal pathway [21]. Moreover, the higher consumption of other sugars when enzyme is added might be related to the higher amount of free glucose in the medium. Davis et al. [3] have noticed a higher consumption of xylose when the stillage medium was supplemented with glucose.

The amount of enzyme added during hydrolysis of lignocellulose-based or derived substrates contributes to a large fraction of the process costs and therefore its amount must be minimised. In this work, the effect of the addition of enzyme diminished at gradually higher concentrations since 0.35 ± 0.02, 0.13 ± 0.00, and 0.08 ± 0.00 g ethanol/L/FPU were obtained when 1, 5, and 10 FPU were used, respectively. Therefore, considering the amount of ethanol produced when progressively higher concentrations of cellulase were used, 1 FPU of enzyme/g SS was chosen and used in further studies.

### 3.2. Whole Stillage Fractions Contribution to Ethanol Production

**Figure 3:**
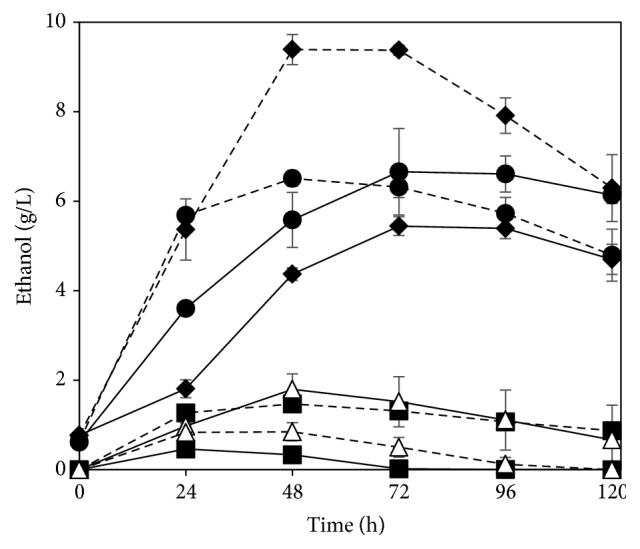
Ethanol production profiles during* N. intermedia* cultivation in undiluted whole stillage (diamonds) and its fractions (supernatant (circles), “small particles” (squares), and “large particles” (triangles)) with (dashed lines) and without (straight line) cellulase addition. Error bars represent two standard deviations.

**Figure 4:**
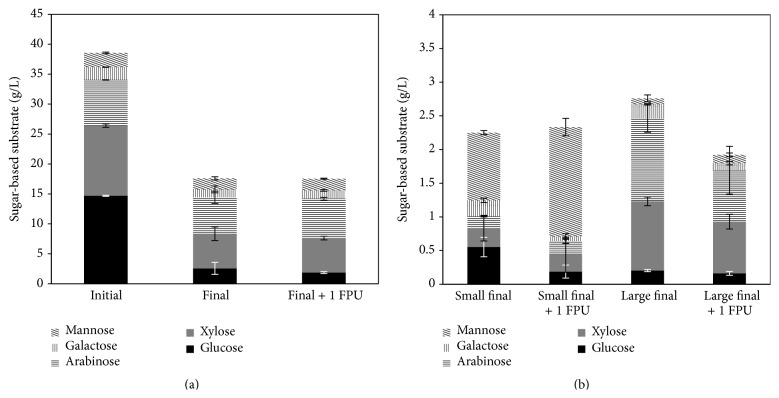
Profiles of dissolved sugar-based substrates after* N. intermedia* cultivation in whole stillage supernatant (a) and small and large particles (b) with addition or not of cellulase. Error bars represent two standard deviations.

Whole stillage is a complex medium in which both solid and liquid fractions contain carbon sources which* N. intermedia* can use to produce ethanol. Therefore, unveiling which fraction contributes the most to the production of ethanol gives an important input towards process understanding of ethanol production from this side stream. Whole stillage was divided into three fractions, namely, sieved solids named “large particles,” solids after centrifugation named “small particles,” and the supernatant. The carbon sources available were around 39 g/L in the supernatant and around 10 g/L and 12 g/L in the large and small particles, respectively. The production of ethanol was studied with addition or absence of 1 FPU cellulase/g SS and the findings are presented in Figure 3. Evidently, the supernatant gave the highest contribution (75%) to the ethanol production, while the ethanol produced from the “large particles” and “small particles” accounted for 20 and 5% of the total amount when no enzyme was added to the medium, respectively. Addition of cellulase led to similar maximum ethanol production, but at a higher production rate (211 ± 6 versus 125 ± 1 mg/L/h) when the supernatant was the cultivation medium. Moreover, no further improvement in the ethanol production from “large particles” was observed, while three times more ethanol was produced when “small particles” were used. An analysis of dissolved saccharides was performed for all different fractions after cultivation with and without enzyme (Figure 4). The most striking differences were the reduction of glucose-based saccharides with a similar reduction of 82–87%, while the reduction of xylose-based saccharides reached 51% when the supernatant was used with or without cellulase. Moreover, higher final concentrations of arabinose- and xylose-based saccharides were obtained when “large particles” were used when compared with those from cultivation with “small particles.” The addition of yeast extract (5 g/L) to the medium containing “large particles” did not lead to further improvements on ethanol production (data not shown).

Interestingly, using just the supernatant as cultivation medium, a higher maximum ethanol production was obtained than that when whole stillage was used as cultivation medium (6.0 versus 4.7 g/L). The reason for the observed behaviour is unknown and so further studies are needed. Moreover, the similar maximum ethanol production when the supernatant was used both with and without addition of cellulase points out the relevant enzymatic capabilities of* N. intermedia*.

### 3.3. Comparison with Yeast

**Figure 5:**
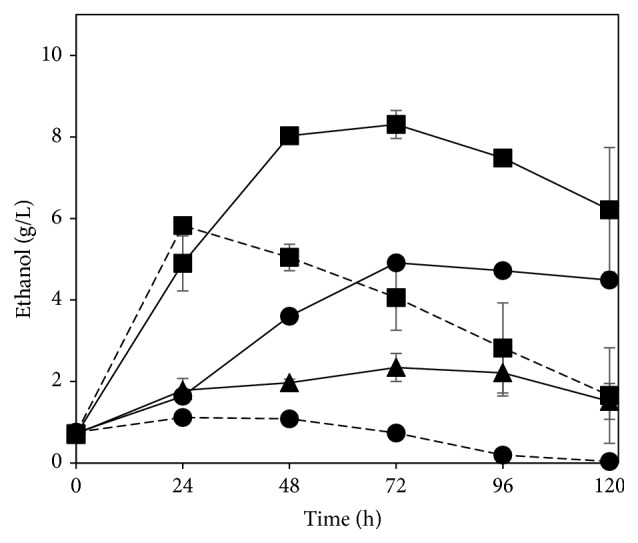
Ethanol profiles during cultivation in whole stillage without cellulase (circles) and with addition of 1 FPU cellulase/g SS (squares) with* N. intermedia* (straight lines) and* S. cerevisiae* (dashed lines) or their coculture (triangles). Error bars represent two standard deviations.

The research with filamentous fungi has been greatly stimulated by their metabolic diversity, easier separation from the medium, and ability to consume pentose sugars, namely, xylose and arabinose, when compared to the baker's yeast [22]. In this work, a performance comparison towards ethanol production between* N. intermedia* and* S. cerevisiae* was carried out and the results are presented in Figure 5. When* N. intermedia* was used as the fermenting agent, higher ethanol production was achieved both in absence of cellulase and when 1 FPU of the enzyme/g SS was used. A more clear difference was observed when no enzyme was added to the medium;* N. intermedia* produced 10.5 times more ethanol than* S. cerevisiae*. Such difference should be related to the higher enzymatic capability of* N. intermedia* to consume and convert glucose- and xylose-based saccharides present in the whole stillage liquid fraction to ethanol. The higher production of ethanol when cellulase was added to the medium might be related to the filamentous fungus ability to convert xylose to ethanol contrary to* S. cerevisiae*. The coculture of both microorganisms also led to a lower amount of ethanol being produced. Therefore, the performance of* N. intermedia* towards ethanol production from whole stillage was clearly superior to that of* S. cerevisiae* pointing out the beneficial potential of the inclusion of* N. intermedia* in the established industrial process of ethanol production.

### 3.4. Two-Stage Cultivation for Production of Ethanol and Fungal Biomass

**Figure 6:**
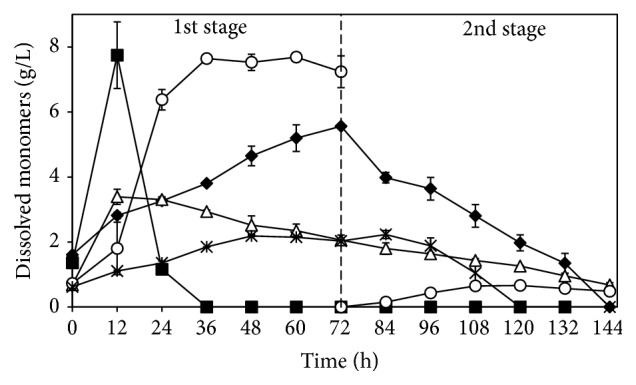
Concentration profiles of arabinose (diamonds), ethanol (circles), glucose (squares), and xylose (triangles) during a two-stage cultivation in a 2.5 L continuous-stirred tank reactor. The first 72 h stage corresponds to the production of ethanol by* N. intermedia* with 1 FPU cellulase/g SS and the second 72 h stage corresponds to the production of biomass by* A. oryzae* after the medium had been distilled and its solids sieved. Error bars represent two standard deviations.

The production of ethanol with addition of 1 FPU cellulase/g SS was also studied using a 2.5 L bench-scale continuous-stirred tank reactor. However, an innovative two-stage cultivation process in order to produce fungal biomass in addition to ethanol was investigated. During the first stage of the process, the maximum ethanol production of 6.9 ± 0.1 g/L was achieved after 36 h and the maximum production rate of 235 ± 13 mg/L/h was reached after 24 h of cultivation with* N. intermedia* (Figure 6). Therefore, the value of ethanol produced in the bioreactor was somewhat lower (6.9 versus 8.7 g/L) than that when cultivation was performed in shake-flasks which indicates that aeration and mixing optimization is needed. During the three-day cultivation, the amount of xylitol increased 233%, the amounts of glycerol and lactic acid did not change significantly, and acetic acid had been totally consumed after 24 h. The main monomeric sugars, arabinose, glucose, and xylose, exhibited different concentration patterns during the first stage of cultivation: the amount of arabinose increased continuously during cultivation, the maximum amount of glucose (7.7 g/L) was measured after 12 h and was depleted after 36 h of cultivation, and xylose increased by 386% after 12 h and 49% of it had been consumed at the end of the first cultivation stage.

**Figure 7:**
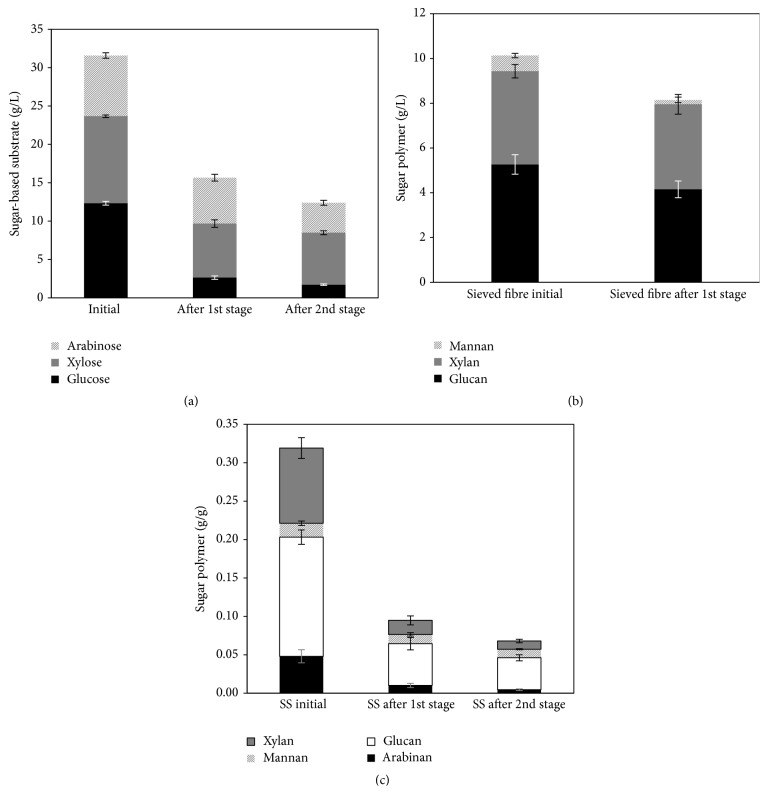
Sugar profiles from the supernatant (a), sieved fibres (b), and suspended solids (c) in a two-stage whole stillage cultivation in a bench-scale reactor. Error bars represent two standard deviations.

The use of the bulk medium whole stillage does not allow fungal biomass to be separated from other medium components. Therefore, the second stage of the process was preceded by a harvesting stage in addition to the evaporation of the medium ethanol and* Aspergillus oryzae* which was used for production of biomass. After 72 h of cultivation, 5.8 ± 0.8 g/L of biomass containing 42.3 ± 1.7% crude protein on a dry weight basis was obtained. Besides, up to 0.7 g/L of ethanol was produced and total consumption of arabinose and xylitol as well as 63% of the xylose was achieved by the end of the cultivation. The lactic acid and glycerol concentrations were reduced by 13 and 9%, respectively.* A. oryzae* was chosen for this second step of the process based on its outstanding capacity to consume glycerol from thin stillage [11]. The low reduction of glycerol might be related to C/N ratio or other more preferable carbon sources for the ascomycete fungus. An analysis of dissolved saccharides and sugar polymers in the liquid and solid fraction, respectively, was also conducted in this part of the study and the main changes are represented in Figure 7. After the two-stage cultivation, the arabinose-, glucose-, and xylose-based saccharides had been reduced by 86, 51, and 40% (w/v), respectively, while the sugars polymers arabinan, glucan, mannan, and xylan present in the suspended solids had been reduced by 91, 73, 38, and 89% (w/v), respectively. The fibres recovered by sieving after the first stage of cultivation had their glucan, mannan, and xylan content reduced by 21, 72, and 9% (w/v), respectively. The spent stillage after the second stage of cultivation contained 8.3 ± 0.4 and 0.9 ± 0.1% (w/v) total and suspended solids, respectively. Cultivation of* N. intermedia* in semisynthetic medium containing single carbon sources corroborated the assimilation of more complex substrates observed in whole stillage. The ascomycete fungus was able to produce ethanol when the sugar polymers Avicel, starch, and xylan were used as cultivation substrate. Maximum ethanol productions of 0.4 ± 0.1 after 144 h and 3.0 ± 0.2 g/L after 48 h were obtained during cultivation in xylan and starch, respectively. Ethanol production from Avicel was slower and after 166 h of cultivation 1.2 ± 0.2 g/L had been produced.* N. intermedia* was also able to consume fully cellobiose after 60 h where the maximum ethanol yield of 0.15 g/g was reached.

Research work with whole stillage towards either production of fungal biomass or ethanol is absent in the scientific literature. However, its centrifuged product thin stillage originated from corn-based ethanol processes has been evaluated for production of fungal biomass. Mitra et al. [23] have obtained up to 20 g/L of biomass containing 46% oil using 6% solids thin stillage. Rasmussen et al. [10] have also produced biomass from thin stillage containing 43% protein and it also contained essential amino acids to humans. Biomass composed of 46% protein has also been produced from molasses-vinasse containing 5% solids [24]. All these research works have pointed out the important role of the microorganism for reduction of the organic matter in the final spent medium.

Altogether, after a two-stage cultivation, 7.6 g/L of ethanol and 5.8 g/L of biomass containing around 42% (w/w) crude protein were produced. Moreover, throughout the present study both filamentous fungi showed their self-ability to degrade more complex substrates enzymatically unaided. In a process inclusion context, the produced ethanol could simply be sent back to the process together with the condensate, while the degradation and assimilation of carbon sources of the whole stillage can have an important positive impact on evaporation and drying costs of the industrial process.

## 4. Conclusions

The valorisation of whole stillage towards ethanol production was improved by addition of cellulase; more 4 g/L of the alcohol was obtained with addition of 1 FPU enzyme/g SS. By applying an innovative two-stage cultivation with* N. intermedia* and* A. oryzae*, 7.6 g/L of ethanol and 5.8 g/L of biomass containing around 42% (w/w) crude protein were obtained. Both filamentous fungi were able to degrade complex substrates in the medium such as arabinan, xylan, and glucan which together with those carbon sources assimilated in the liquid fraction will potentially have a positive impact on evaporation and drying costs of the industrial process.* N. intermedia* was also shown to be superior to* S. cerevisiae* regarding ethanol production from whole stillage both with and without addition of cellulase to the medium.

## Figures and Tables

**Table 1:** Characteristics of the mostly wheat-based stillage used in the study.

Parameter	Value	Dissolved monomers (g/L)		Dissolved saccharides (g/L)^c^		Sugar polymers (g/L)^d^	
		Parameter	Value	Parameter	Value	Parameter	Value
pH	4.3 ± 0.0	Acetic acid	0.4 ± 0.1	Arabinose	6.3 ± 0.1	Arabinan	1.8 ± 0.1
Total solids (% w/w)	15.6 ± 0.1	Arabinose	1.6 ± 0.1	Galactose	1.7 ± 0.0	Galactan	0.3 ± 0.0
Suspended solids (% w/w)	8.8 ± 0.0	Ethanol	0.7 ± 0.0	Glucose	12.0 ± 0.3	Glucan	4.7 ± 0.1
Sieved solids (% w/v)	3.2 ± 0.2	Glucose	1.4 ± 0.1	Mannose	2.4 ± 0.1	Mannan	0.6 ± 0.0
Crude protein (% w/w)^a^	32.0 ± 0.6	Glycerol	12.0 ± 0.1	Xylose	9.7 ± 0.1	Xylan	3.6 ± 0.1
Crude protein (% w/w)^b^	15.1 ± 3.9	Lactic acid	1.7 ± 0.0				
		Xylitol	0.6 ± 0.1				
		Xylose	0.7 ± 0.1				

^a^Based on dry total solids.

^b^Based on dry sieved solids.

^c^Dissolved monomers included.

^d^From dry sieved solids.
